# Alterations in cell arrangements of group B streptococcus due to virulence factor expression can bias estimates of bacterial populations based on colony count measures

**DOI:** 10.1099/mic.0.001453

**Published:** 2024-04-24

**Authors:** Ruby Thapa, Kelvin G. K. Goh, Devika Desai, Ellen Copeman, Dhruba Acharya, Matthew J. Sullivan, Glen C. Ulett

**Affiliations:** 1 School of Pharmacy and Medical Sciences and Menzies Health Institute Queensland, Griffith University, Gold Coast Campus, QLD 4222, Australia; 2 School of Biological Sciences, University of East Anglia, Norwich, NR4 7TJ, UK

**Keywords:** bacteria, bacterial population estimates, cell arrangement, chain, colony-forming unit

## Abstract

Group B streptococcus (GBS) is a chain-forming commensal bacterium and opportunistic pathogen that resides in the gastrointestinal and genitourinary tract of healthy adults. GBS can cause various infections and related complications in pregnant and nonpregnant women, adults, and newborns. Investigations of the mechanisms by which GBS causes disease pathogenesis often utilize colony count assays to estimate bacterial population size in experimental models. In other streptococci, such as group A streptococcus and pneumococcus, variation in the chain length of the bacteria that can occur naturally or due to mutation can affect facets of pathogenesis, such as adherence to or colonization of a host. No studies have reported a relationship between GBS chain length and pathogenicity. Here, we used GBS strain 874391 and several derivative strains displaying longer chain-forming phenotypes (874391p*gapC,* 874391Δ*covR*, 874391Δ*stp1*) to assess the impact of chain length on bacterial population estimates based on the colony-forming unit (c.f.u.) assay. Disruption of GBS chains via bead beating or sonication in conjunction with fluorescence microscopy was used to compare chaining phenotypes pre- and post-disruption to detect long- and short-chain forms, respectively. We used a murine model of GBS colonization of the female reproductive tract to assess whether chaining may affect bacterial colonization dynamics in the host during chronic infection *in vivo*. Overall, we found that GBS exhibiting long-chain form can significantly affect population size estimates based on the colony count assay. Additionally, we found that the length of chaining of GBS can affect virulence in the reproductive tract colonization model. Collectively, these findings have implications for studies of GBS that utilize colony count assays to measure GBS populations and establish that chain length can affect infection dynamics and disease pathogenesis for this important opportunistic pathogen.

## Introduction

Group B streptococcus (GBS), also known as *Streptococcus agalactiae*, is a Gram-positive, chain-forming commensal bacterium that resides in 30–40 % of adult humans, typically within the gastrointestinal tract and/or vagina [1,2]. It is an important opportunistic pathogen that affects 4 10 000 people annually, causing various types of infections in neonates, pregnant women, nonpregnant adults and the elderly and immunocompromised [3,4]. Globally, maternal GBS prevalence has been estimated to be 18 %, with approximately half of colonized mothers transmitting GBS to their infants before or during childbirth [3,5,7]. In pregnant women, GBS can cause several complications, including intrapartum fever, premature rupture of membranes, preterm delivery, and systemic maternal GBS disease [5]. GBS can be transmitted from mothers to infants before or during childbirth, which can cause neonatal sepsis, meningitis, and encephalopathy [6,8,10]. It has been suggested that 3.5 % of all cases of neonatal death are a consequence of GBS infection [10,11]. GBS is also an important pathogen of nonpregnant adults, especially immunocompromised individuals and the elderly [11,12]. Adults over the age of 70 are more susceptible to GBS-associated pneumonia and urinary tract infections [13].

The mechanisms by which GBS transitions from a frequent colonizer of healthy adults into a potent pathogen are based in part on the expression of various virulence factors that affect host–pathogen interactions and support bacterial survival. Among an array of virulence factors of GBS that support pathogenesis are two-component systems (TCSs), including CovR, phosphatase–kinase pairs such as Stp1 that affect virulence [14,15], cytotoxins, antiphagocytic capsular polysaccharide, enzymes that subvert host immune responses, and adhesins for colonization. Glyceraldehyde 3-phosphate dehydrogenase (GAPDH) is an example of a surface-expressed GBS enzyme that functions in pathogenesis. Encoded by *gapC*, it mediates binding to host extracellular matrices, modulates innate immunity, supports resistance to reactive oxygen species, and colonization [2,16,20].

Streptococci can form chains of variable length [21], with such variation related to strain distinction, particular genetic mutations [22,23], and being linked with virulence. Chaining has been studied in pneumococcus [24], *S. mutans* [25], *S. sanguinis* [22], and *S. pyogenes* [26]. In *S. pneumoniae*, longer chains support colonization and adhesion [23,24, 27]. Long chaining in *S. mutans* ablates phagocytic efficiency [25]. In GBS, longer chaining has been observed in strains lacking *stp1* [15], overexpressing GAPDH [20], and mutated for *covR* (unpublished observations, M.J. Sullivan) [28]. These factors that have been associated with altered chain length in GBS are related to virulence; CovR, Stp1 and GAPDH all influence virulence in GBS. Thus, chain length in streptococci is affected by distinct virulence factors [23,24, 27].

A commonly used method to estimate the number of viable bacteria in a population is the colony count method that is widely applied to study GBS virulence. In studies where a difference in strain background or targeted mutation causes altered chaining, it is conceivable that altered chain length may influence estimates of bacterial colony-forming units (c.f.u.) that could bias comparisons of population estimates. A chain of 50 cocci would give rise to 1 colony, but so would a single viable coccus; in this way, longer chains might cause an underestimate of a population and confound studies comparing bacterial strains with divergent chaining phenotypes. While studies of various *Streptococcus* spp. recognize that chain length can influence pathogenesis, no studies have investigated the impact of chain length on c.f.u. estimates for GBS. Here, we analysed GBS 874391, a representative serotype III, sequence type 17 hyperinvasive strain isolated from the human female genital tract [29] and derivative strains related to expression of GAPDH, or isogenic mutants in *covR* and *stp1* to assess whether variable chain length of GBS might bias population estimates from c.f.u. assays.

## Methods

### Bacterial strains, culture conditions and population estimates

GBS 874391 [29] and 874391p*gapC*, a derivative that overexpresses GAPDH from p*gapC* [a chimeric construct of *gapC* fused to the constitutive ‘on’ promoter for *ahpA3* encoding kanamycin resistance [18] in the streptococcal shuttle plasmid pDL278 that carries a spectinomycin (Sp) resistance (Sp^R^) gene] were used with a control strain carrying pDL278. Additional GBS strains used in this study were 874391*ΔcovR*, and 874391Δ*stp1* and its control strain 874391Δ*stp1::*p*stp1/k1* [28,30] (Table 1). Bacteria were grown at 37 °C on horse blood agar [HBA; tryptone soya broth (OXOID CM0129), 1.5 % bacteriological agar (OXOID, LP0011) +5 % defibrinated horse blood (OXOID, SR0050) or in Todd Hewitt broth (THB, OXOID, CM0189) with shaking at 200 r.p.m. overnight; typically, these were 10 ml THB cultures with supplemental Sp (100 µg ml^−1^) as required. Colony counts were used for c.f.u. estimates using serial dilutions on agar incubated overnight.

**Table 1.** Bacterial strains and plasmids used in this study

Bacterial strains	Characteristics^*^	Reference
*S. agalactiae* 874391	Wild-type, sequence type 17, serotype III strain	[37,38]
*S. agalactiae*GU2852	*S. agalactiae* 874391 overexpressing GAPDH (carrying pDL278*::gapC*); Sp^R^	[20]
*S. agalactiae*GU2672	*S. agalactiae* 874391 carrying pDL278 (empty vector control strain); Sp^R^	[20]
*S. agalactiae* GU3055	874391*Δstp1* (*stp1* mutant)Locus tag: CHF17_00435	[30]
*S. agalactiae* GU3126	874391*Δstp1* pGU3119 (pDL278::*stp1/k1*)	[30]
*S. agalactiae* GU2400	874391*ΔcovR* (*covR* mutant); Cm^R^Locus tag: CHF17_01656	[28]
**Plasmids**		
pDL278	Shuttle vector for complement studies; Sp^R^	[39]
pGU3119	pDL278*::stp1/k1* complement construct, Sp^R^	[30]

*SpR, spectinomycin-resistant; CmR, chloramphenicol resistant; Ts, temperature-sensitive.

### Disruption of GBS chains

We used two separate methods based on bead beating and sonication to disrupt longer chains of GBS prior to undertaking c.f.u. estimates. Bead beating is commonly used in studies of bacterial virulence to generate sample (such as tissue) homogenates prior to plating for c.f.u. estimates, and sonication is an accepted method to disrupt streptococcal chains. Cultures were centrifuged (Thermo Fisher Scientific Heraeus Multifuge X3R, F13−14×50 CY) at 8000 ***g*** for 15 min at 20 °C, the supernatant was discarded, and the pellet was washed and resuspended in sterile 1× phosphate-buffered saline (PBS) (pH 7.4) prior to bead beating. One ml of suspension of culture was transferred to a 2 ml microfuge tube containing two sterile stainless-steel beads (3 mm). The suspension was beaten for 1 min at 30 f/s (Qiagen Tissue Lyser II). The beating was repeated, and the samples were then serially diluted and plated on HBA for c.f.u. counts.

For sonication, overnight cultures were centrifuged at 8000 ***g*** for 15 min at 20 °C. The pellet was washed and resuspended in PBS. A Sonics Vibra Cell (John Morris Scientific) with a 1/8 inch probe was used to sonicate the cells at 30 % amplitude, 15 s pulse on/off, and a time of 15 s at 22 °C. The samples were held on ice during sonication and were serially diluted for colony counts on HBA. For both bead beating and sonication assays, samples of the same culture were assayed pre- and post-disruption to determine the direct effect of the disruption on c.f.u. estimates of a single culture. Disruption experiments were repeated at least four times independently using identical conditions.

### Fluorescence microscopy

To visualize bacterial chaining pre- and post-disruption by bead beating and sonication we used fluorescence microscopy; 15 µl of bacterial samples was spotted onto a slide. Samples were dried on a heat block for 15 min set to 42 °C. The cells were then fixed for 10 min at 4 °C with 30 µl of 3.5 % PFA and two washes were performed with sterile PBS. The cells were stained with 15 µl of Hoechst (Solution 33 342, Thermo Fisher Scientific) and the slide was then incubated at 37 °C for 10 min. Following three water washes, samples were dried at room temperature, and 15 µl of mountant was applied, prior to coverslip and sealant with varnish and incubated at 37 °C for 30 min. Samples were analysed using an AxioImager.M2 Microscope (Carl Zeiss MicroImaging) fitted with Plan-Apochromat x63/1.40 and x100/1.46 objective lenses. We used Zen SP2 imaging software for image acquisition. ImageJ software (https://imagej.net/software/fiji/) was used to measure the area of the bacterial cells in chains based on images that were captured using the x63 objective lens; the average total pixel counts for 30 chains were compared.

### Animal studies

Wild-type C57BL6/J female mice, 6–8 weeks old, were purchased from the Animal Resource Centre, Western Australia. Overnight cultures of 874391, 874391p*gapC*, 874391Δ*covR*, and 874391Δ*stp1* were grown in THB, washed twice with sterile PBS, and suspended in 1 ml PBS. Each resuspended inoculum was divided into two equal samples (with each containing equivalent bacterial biomass), labelled ‘pre-disruption’ and ‘post-disruption’; one of the samples was subjected to bead beating as above to disrupt the bacterial chains. Each sample was quantified by c.f.u. estimates prior to inoculation of mice. A single sub-cutaneous injection of 50 µl of 17β-estradiol valerate (Sigma, E1631-1G), prepared as follows, was given to all mice to synchronize all animals in the oestrous cycle prior to inoculation; 10 mg estradiol was dissolved in 200 µl 100 % ethanol (T038181000, Sigma, T038181000) and vortexed until dissolved, then 4800 µl castor oil (Sigma, 259853) was added and mixed prior to use. The mice vaginal vaults were flushed with 0.2 % Triton X-100 (93 443, Sigma) in sterile water, filter-sterilized using a 2 mm filter (Millex-HV, Merck) followed by PBS 1 h prior to inoculation. Each mouse was challenged with 10 µl of pre-disrupted or post-disrupted bacterial sample, with each sample containing the same bacterial biomass. We collected vaginal swabs on D1 post-infection (p.i.) and then subsequently on every 3rd day until D28. On the 28th day, vaginal swabs were collected, and mice were euthanized; uterine horns were collected, homogenized, and serially diluted for colony counts. Swab samples were serially diluted in sterile PBS and plated on HBA, ColNAC, THA-SPEC, and CHROMagar to provide population estimates of total bacteria, Gram-positive bacteria, pDL278-carrying GBS, and GBS specifically. Ten mice per group were used for each experiment that were repeated four independent times. Data shown represent the pooled data of all experiments.

### Statistical analysis

Data were analysed and compared using SPSS v29 and GraphPad v10.1.0. Specific comparisons were area-under-the-curve (AUC), Mann–Whitney U-tests, independent sample *t*-tests, paired *t*-tests, and Fisher’s exact tests, as specified in respective the figure legends. Statistical significance was accepted as *P*<0.05.

## Results

### Overexpression of GAPDH causes long-chaining in GBS that can be disrupted mechanically

**Fig. 1.**
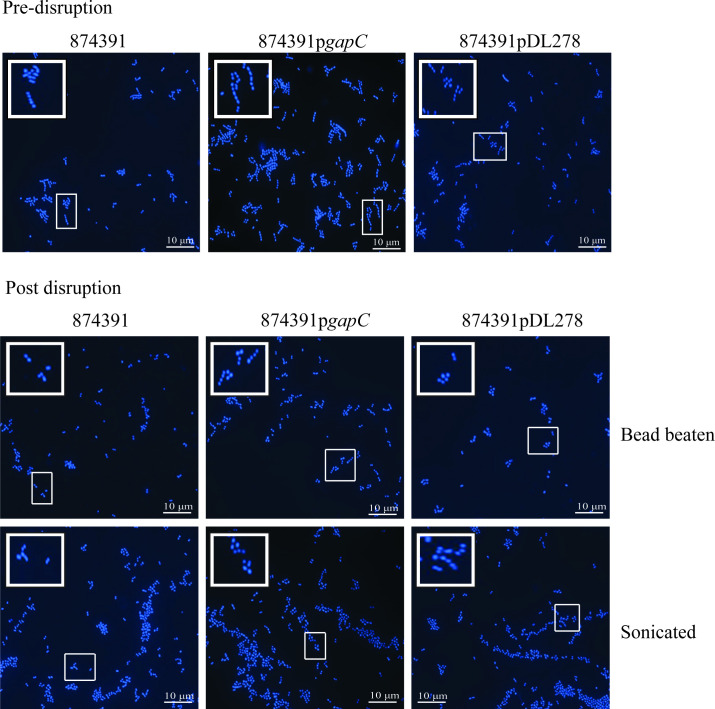
Chaining phenotype of 874391, 874391p*gapC*, and 874391pDL278 cells. Overnight cultures of the three strains before and after disruption were stained with HOECHST and visualized under a microscope. Inserts depict blown-up sections of the slide boxed in white. Images were taken using a X100 objective lense, scale bars=10 µm.

Initially, we sought to confirm long-chaining in GBS 874391 overexpressing GAPDH, as reported previously [20]. For this, we stained overnight cultures of wild-type (WT) 874391, 874391p*gapC* (overexpresses GAPDH) and 874391pDL278 (empty vector control) with Hoechst and visualized the strains using fluorescence microscopy prior to any mechanical disruption to break chains. This confirmed a phenotype of longer chaining and clumping in 874391p*gapC* versus WT and 874391pDL278 for which chaining phenotypes were similar between the strains (Fig. 1; Pre-disruption).

We next examined the effect of mechanical disruption of the chains of GBS using two methods to physically disassociate the cells constituting chains – bead beating and sonication. To visualize the effect of bead beating and sonication on chain length, bacterial samples were stained as above and examined using fluorescence microscopy. The relatively abundant shorter chains of WT 874391 and 874391pDL278 (compared to 874391p*gapC*) were effectively disrupted into single cocci and diplococci by bead beating or sonication (Fig. 1; post-disruption). The effect of mechanical dissociation of cells from chains was amplified in 874391p*gapC*, where bead beating, or sonication caused disruption of the long chains of this strain (Fig. 1; post-disruption). While bead beating or sonication disrupted the chains of GBS, more clumping was apparent after sonication. Analysis of microscopy images using ImageJ to quantify chaining (i.e*.* as an average cellular area per chain/cluster based on fluorescent pixels) showed significant reductions in the sizes of chains/clusters post-disruption for WT 874391, 874391p*gapC* and 874391pDL278; among these strains the magnitude of the effect of disruption was most notable for 874391p*gapC* (Table 2).

**Table 2.** Average chain area of GBS strains pre- and post-disruption by bead beating

	Chain area (average pixel count per chain, mean±sd) ^*^					
	**WT 874391**	**874391p*gapC***	**874391pDL278**	**874391Δ*covR***	**874391Δ*stp1***	**874391Δ*stp1*:: p*stp*/*k1***
Pre-disruption	17.16 (±14.83)	53.10 (±27.03)	25.23 (±13.74)	52.11 (±30.43)	533.40 (±352.3)	3.20 (±1.97)
Post-disruption	11.46 (±5.73)	14.16 (±8.51)	13.23 (±6.87)	9.26 (±8.52)	15.76 (±5.91)	2.17 (±1.82)
*P* value	0.029	<0.001	<0.001	<0.001	<0.001	0.020

*Mean area of chains was measured using ImageJ software with images captured at captured using a Plan-Apochromat x63/1.40 objective lens ; the average (fluorescence-emitting) pixel count for thirty30 chains was determined for each strain pre- and post-disruption by bead beating and compared using paired Student’s *t*-tests.

### Quantitation of the disruption of GBS chains caused by beat beating or sonication

**Fig. 2.**
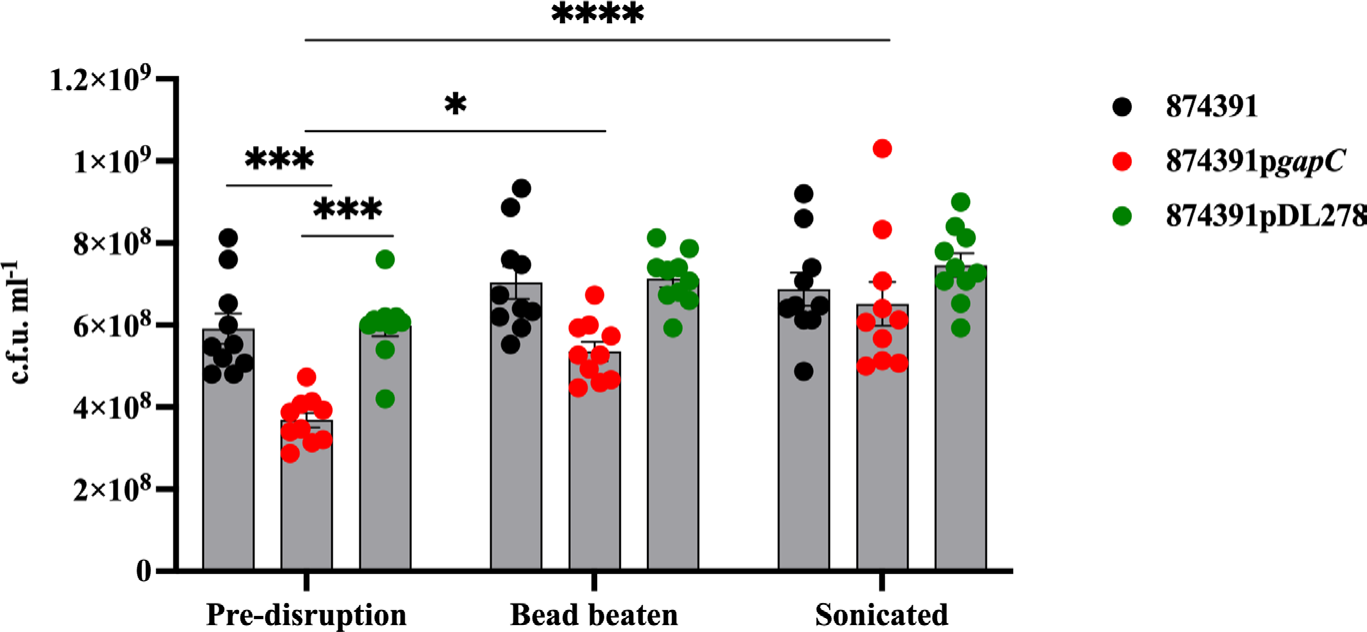
Colony count measures of 874391, 874931p*gapC*, and 874391pDL278 pre- and post-disruption. Overnight cultures pre- or post-disruption (beat beating or sonication) were plated on HBA to study the effect of disruption on population estimates. Counts were compared using a Kruskall–Wallis test with Dunn’s multiple comparisons, with error bars indicating sem (*n*=10; *, *P*<0.04, ***, *P*<0.002, ****, *P*<0.001).

To determine whether chaining phenotypes of GBS might bias estimates of population size based on colony counts, we grew overnight cultures of WT 874391, 874391p*gapC* and 874391pDL278 and compared the relative culture yields according to c.f.u. pre-disruption and post-disruption by bead beating or sonication. Colony count measures were significantly higher for WT and 874391pDL278 versus longer chain-forming 874391p*gapC* (Fig. 2). Notably, when measured pre-disruption using absorbance readings at OD_600nm_ the culture densities of the three strains were similar (Fig. S1, available in the online version of this article). These findings suggested that the total cell biomass of each separate culture was similar despite significant differences in measures of c.f.u. pre-disruption. Examination of the effect of mechanical disruption of chains on c.f.u. counts revealed a significant increase in recovery of 874391p*gapC* c.f.u. post-disruption by bead beating or sonication as compared to pre-disruption (Fig. 2; compare red circles pre-disruption 874391p*gapC* and bead beaten or sonicated 874391p*gapC*). Conversely, WT 874391 and 874391pDL278 showed no statistically significant difference in recovery of c.f.u. post-disruption (Fig. 2). Together, these results show that mechanical disruption of chains significantly increases GBS c.f.u. estimates for some strains such as 874391p*gapC* that form longer chains relative to other strains.

### Mutations in GBS TCS genes cause elongated chaining that can be disrupted by beat beating

**Fig. 3.**
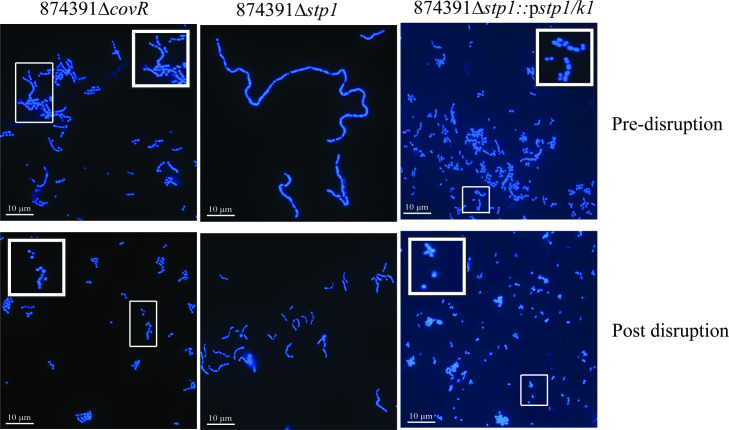
Chaining phenotype of 874391ΔcovR, 874391Δ*stp1*, and 874391Δ*stp1*::p*stp1/k1* cells. Overnight cultures of the strains pre- and post-disruption were stained with HOECHST and visualized under a microscope. Insets show enlarged views of the smaller white boxed areas. Images were taken using a X100 objective lense, scale bars=10 µm.

**Fig. 4.**
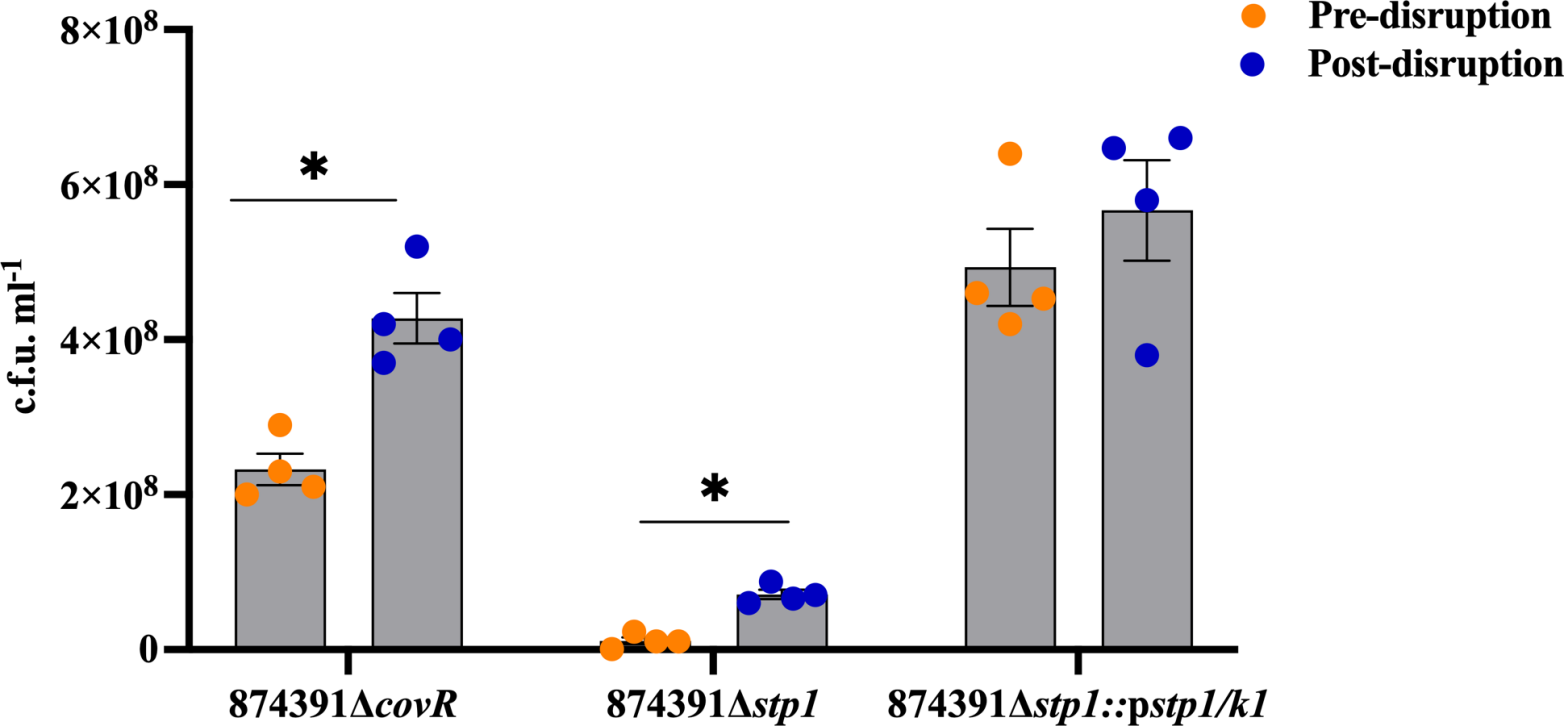
Colony count measures of 874391Δ*covR*, 874391Δ*stp1*, and 874391Δ*stp1*::p*stp1/k1* pre- and post-disruption. Cultures pre- or post-disruption were plated on HBA to study the effect of disruption on c.f.u. counts. Counts pre- and post-disruption were compared using a Mann–Whitney U test, with error bars indicating sem (*n*=4; *, *P*<0.05).

To determine the relevance of our findings on chain length and c.f.u. estimates to studies that utilize GBS mutants deficient in gene(s) for virulence factors or regulatory systems, we used GBS 874391 isogenic mutants previously reported to form long chains, namely 874391Δ*covR* and 874391Δ*stp1* (along with its complement strain 874391Δ*stp1::*p*stp1/k1*). Firstly, we prepared overnight cultures and assessed these using fluorescence microscopy both before disruption and after beat beating (post-disruption). As expected, 874391Δ*covR* and 874391Δ*stp1* exhibited long-chain phenotypes, which were dissociated into shorter chains post-disruption (Fig. 3). While bead beating disrupted long chains of 874391Δ*stp1*, it did not generate purely single cocci or diplococci. This effect was less prominent in 874391Δ*stp1::*p*stp1/k1*. Next, we measured c.f.u. counts on the TCS mutants pre- and post-disruption to assess whether the disruption of longer chains caused an increase in recovery of c.f.u. for the respective strain post-disruption. Here, we observed a significant increase in c.f.u. recovery post-disruption for 874391Δ*covR* and 874391Δ*stp1* (Fig. 4). A higher mean c.f.u. recovery for 874391Δ*stp1::*p*stp1/k1* comparing post-disruption and pre-disruption was not statistically significant. Notably, 874391Δ*stp1*, which exhibited the most elongated chains of any strain, were recovered in significantly lower c.f.u. pre-disruption as compared to the other strains (Fig. 4).

In surrogate measures of chain size using average number of fluorescent pixels comprising distinct separate chains, chains of GBS TCS mutants with long-chaining phenotypes exhibited significantly higher pixel counts prior to chains being disrupted by bead beating, with the magnitude of the effect most notable for 874391Δ*stp1* (Table 2). Finally, we also observed that this effect can relate not only to long-chains, but also to clumps/aggregates of chains and cells that contribute to underestimating population measures depending on the GBS strain. For example, higher population measures for 874391Δ*stp1* are due exclusively to long-chaining (Fig. 4), but both chains and clumps/aggregates contribute to higher population measures for 874391Δ*covR* (Fig. 3) and 874391p*gapC* (Fig. 1), which form both these cell arrangements (Table 3). Taken together, these observations show that disruption of GBS chains and clumps/aggregates by bead beating significantly increases population estimates, depending on the cell arrangements formed by a given GBS strain.

**Table 3.** Proportion of chains versus clumps in cell arrangements of GBS strains in this study

	WT 874391	874391p*gapC*	874391pDL278	874391Δ*covR*	874391Δ*stp1*	874391Δ*stp1*:: p*stp1*/*k1*
Chains (*n*)^*^	113±76	120±66	133±85	69±36	17±12	87±40
Clumps (*n*)^*^	12±4	15±9	15±5	7±4	1±1	9±4
Proportion of chains (%)	82.3±13.9	85.7±8.3	86.2±5.0	89.5±1.4	94.1±5.0	86.8±9.4

*Numbers of chains and clumps were counted in three fields of view captured atusing x63 and x100 objective lenses (six fields of view total) for each strain pre-disruption. Data shown represent pooled data of mean counts (*n*±SDsd) for six fields of view and the mean proportion of chains (%±SDsd) in each field of view.

### Mechanically disrupting long chains of GBS can affect bacterial colonization *in vivo*

**Fig. 5.**
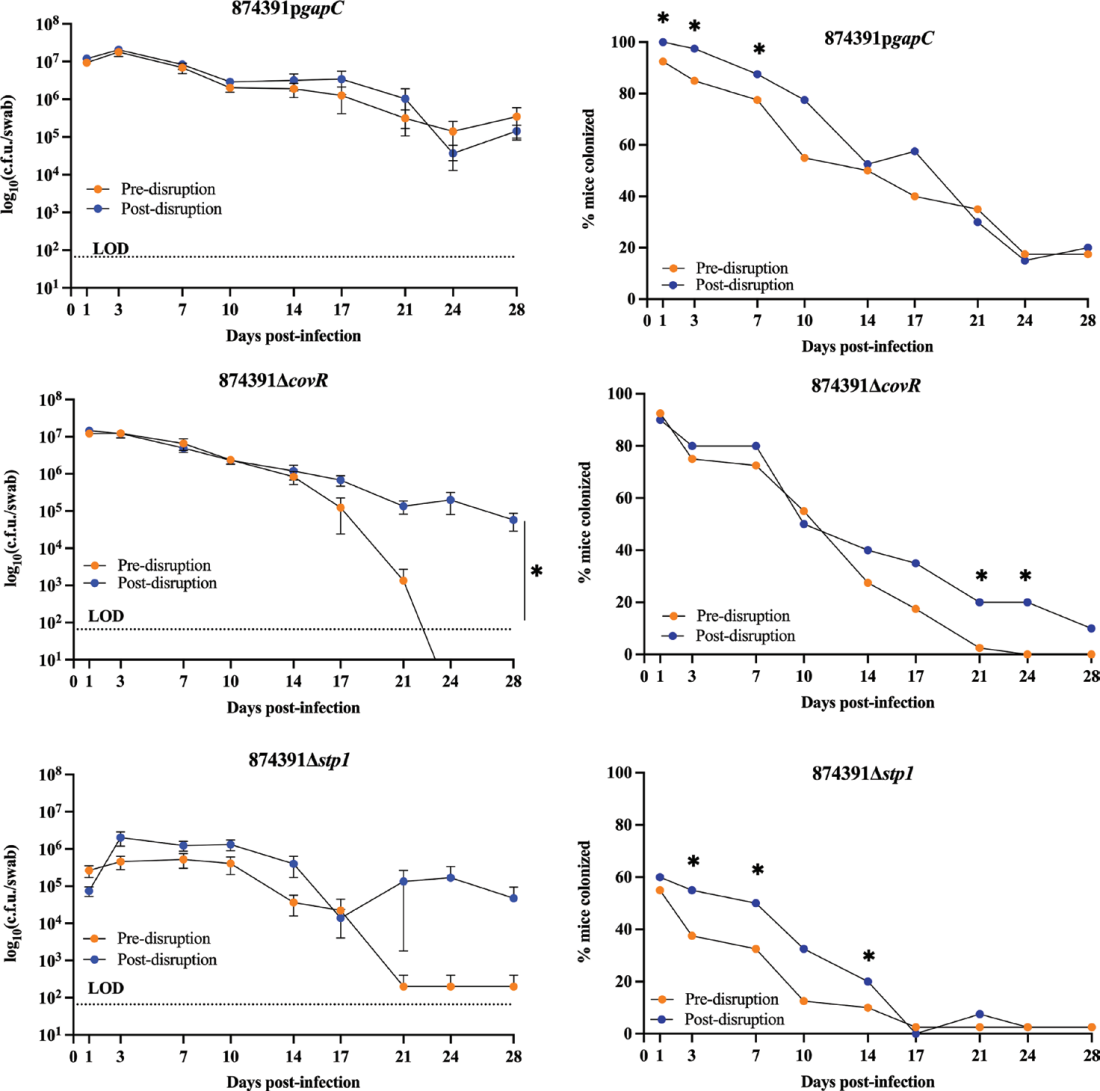
Effect of chain length of 874391p*gapC*, 874391Δ*covR*, and 874391Δ*stp1* in a mouse model of reproductive tract colonization. Six-to-8 week-old female C57BL6/J mice were inoculated with either pre- or post-disrupted 874391p*gapC*, 874391Δ*covR*, and 874391Δ*stp1* using a 10 µl inoculum delivered to the vaginal vault. (**a**) Swab samples were collected every 3–4 days p.i. and plated onto CHROMagar to visualize the GBS colonies and determine bacterial load. The data shown represent pooled c.f.u. counts from four experiments, and error bars represent the standard error of the mean. The limit of detection (LOD) is 66 c.f.u. The area under the curve was calculated for all samples from D14-D28 p.i. and compared with a Student’s *t*-test (*n*=38–40; *, *P*<0.05). (**b**) The percentage of mice in each group that exhibited culture positive status for CHROMagar. The number of colonized mice pre-and post-disruption at each time point were compared using a Chi-square test (*, *P*<0.05).

Given the effect of GBS chaining on population estimates based on c.f.u. measures that are used routinely as a technique to study GBS pathogenesis, we next investigated whether disruption of long chains of GBS could affect host colonization. Using a murine model of reproductive tract colonization, we inoculated mice with pre- and post-disrupted 874391p*gapC*, 874391Δ*covR*, and 874391Δ*stp1*, and monitored GBS load over time. Each strain efficiently colonized all mice at D1 p.i., allowing comparisons of c.f.u. loads based on equivalent early infection. We observed no differences in colonization for 874391p*gapC* between pre- and post-disruption groups over time, according to bacterial load (c.f.u.; Fig. 5, left), but the proportion of mice colonized (according to culture-positive status; Fig. 5, right) differed significantly, with post-disruption 874391p*gapC* colonizing more mice between D1-D14 p.i. versus 874391p*gapC* pre-disruption. For 874391Δ*covR*, significantly more c.f.u. were recovered from D14 p.i. onwards in the post-disruption group as compared to the pre-disruption group, with higher mean recovery from D17 p.i. onwards (c.f.u.; Fig. 5, left). There was also higher mean recovery of post-disruption 874391Δ*stp1* as compared to the pre-disruption group from D21 p.i. onwards (c.f.u.; Fig. 5, left), although these differences did not reach statistical significance according to comparisons of AUC due to high variation among the groups across independent experiments. The proportions of mice colonized with 874391Δ*covR* and 874391Δ*stp1* were significantly higher for the post-disruption group as compared to the pre-disruption group for each respective strain (Fig. 5, right). Together, these data show that mechanical disruption of long chains of GBS TCS and phosphatase–kinase mutants can significantly affect the degree of bacterial colonization of a host.

## Discussion

*Streptococcus* spp. divide in one plane and thus occur in pairs as diplococci, or in chains of varying length, especially in liquid media or clinical material [31]. Different strains of one species can also form chains of variable length [21], which has been associated in some instances with gene mutation [22,23]. Additionally, chain length in streptococci has been studied in relation to virulence of a few species, including pneumococcus [24], *S. mutans* [25], *S. sanguinis* [22], and *S. pyogenes* [26], contributing to complement evasion, adherence to host cells, and biofilm formation. For GBS, it is unclear whether chain length can affect virulence or disease pathogenesis. Among the most routinely used tools to study GBS and other streptococcal virulence is the colony count assay, used to estimate bacterial population size and compare distinct infection or treatment groups. In this study, we examined whether altered chain length of GBS might (i) influence estimates of bacterial population size based on c.f.u. measures and thereby bias results of such measures, and (ii) affect virulence or pathogenesis *in vivo* independent of bias related to assay artefact due to chain length. The main findings of this study are that GBS chain length can bias population size estimates from c.f.u. measures for strains that form long chains, implying that chain disruption before colony count assays is necessary to generate accurate population size estimates. Additionally, GBS chain length can affect virulence *in vivo*, including colonization efficiency within the female reproductive tract.

Complex mechanisms are involved in cellular chain length for streptococci; cell wall components such as peptidoglycan, lipoteichoic acids, and cell wall anchor proteins affect the morphology of cells [32], and multiple genes have been associated with differences in chain length [22]. Some genes that are linked with altered chaining in bacteria are also involved in the ability of cells to divide and separate, with those encoding TCSs regarded as important [22]. We used several strains of GBS to examine chaining in this study, including 874391p*gapC* that overexpresses the sticky surface-located protein GAPDH [2,16,20], and mutants in the TCSs Stp1 [15] and CovR [28]. In studying these GBS strains for c.f.u. yield in *in vitro* assays following disruption of chains by mechanical means, we observed significantly higher yields for each long-chain form that was subjected to bead beating or sonication, with microscopy used to confirm disruption of chains. Microscopy data using measures of chain length based on fluorescent pixels showed consistency with c.f.u. estimates in demonstrating significantly longer chains in the TCS mutants compared to the WT. The increases in c.f.u. yield included higher recovery of 874391p*gapC* post-disruption compared to 874391p*gapC* pre-disruption, as well as post-disrupted 874391Δ*covR* and 874391Δ*stp1* compared to their pre-disrupted forms. These findings show that population size estimates of GBS strains with long-chaining phenotypes due to overexpression of a variety of genes, including for GAPDH or various genes impacted by GBS TCSs, are significantly affected when the long chains of the bacteria are mechanically dissociated. Taken together, these findings show the importance of disrupting long-chain GBS for population size estimates, especially in studies that compare strains of different chain-forms to avoid bias in these estimates from different chain forms.

The *in vivo* colonization experiments in this study were designed to determine whether chaining differences at the time of inoculation can affect early colonization and/or subsequent infection progression dynamics over time; we compared two groups of mice inoculated with a standard volume of the same inoculum preparation (i.e*.* meaning the same biomass) where one aliquot of the inoculum was subjected to bead beading immediately prior to inoculation of the test group. In this way, the single variable was the disruption of GBS chains prior to inoculation in the test group versus the control (pre-disruption) group, limiting our comparison to within-strain effects (of chain disruption). The results indicate that the ability of GBS to colonize the female reproductive tract in mice can be affected by chain length for immediate/early colonization, but the dynamics of chronic colonization (and proportion of culture-positive mice) can be significantly affected by chain length. For example, shorter-chain 874391p*gapC* (post-disruption) was recovered from a significantly higher proportion of mice (colonized) between D1-D14 p.i. compared to 874391p*gapC* in longer-chain form (pre-disruption). Our interpretation of these data is that mechanical disruption of GBS chains prior to inoculation can increase colonization efficiency *in vivo* and affect infection dynamics *in vivo* for strains such as 874391p*gapC* that form long chains. For 874391Δ*covR* and 874391Δ*stp1*, higher recovery of shorter-chain form (post-disruption) from D14 p.i. compared to longer-chain form (pre-disruption), together with more mice being chronically colonized with shorter- (post-disruption) versus longer-chain (pre-disruption) form support an interpretation that mechanical disruption of long-chain GBS can affect the colonization efficiency of this pathogen in the host environment *in vivo*.

It is also interesting to consider the colonization dynamics of the shorter- (post-disruption) versus longer-chain (pre-disruption) forms of GBS in the mice in this study, noting the differences in recovery of bacteria that occurred early post-inoculation (i.e. compare post-disruption and pre-disruption forms of 874391p*gapC* and 874391Δ*stp1* between D1-D14 p.i., which were significantly different for short versus long-chain GBS); conversely, significant differences were apparent much later in the course of infection for the shorter- (post-disruption) versus longer-chain (pre-disruption) forms of 874391Δ*covR* GBS (i.e. between D21–D28 p.i.). The immune response to infection during this period develops in two major phases in the innate and adaptive response that separated temporally around D10-D14 p.i. where adaptive responses can generate effector activity for specific antimicrobial responses. Whether distinct immune responses account for the different infection clearance dynamics for 874391p*gapC*, 874391Δ*stp1*, and 874391Δ*covR* GBS over the infection periods in this model is unknown. A study limitation of our *in vivo* analysis of shorter- and longer-chain forms of 874391p*gapC* is that we chose to not administer Sp to the mice in view of prior observations of c.f.u. counts of these strains on THA-Sp compared to CHROMagar from similar *in vivo* assays [20] and harmful effects of antibiotics on the microbiota of mice that we sought to avoid. Therefore, some loss of plasmid from both the shorter- and longer-chain forms of 874391p*gapC* in these assays likely occurred due to an absence of Sp selection pressure *in vivo*.

Exactly how does the chain length of streptococci influence virulence in a host environment? Several examples from studies on different *Streptococcus* spp. highlight apparent opposing effects and are useful to consider the complexity of chaining effects. For example, longer chains of *S. pneumoniae* promote colonization and adhesion to host cells [24,27] but are also more readily taken up by neutrophils and tend to activate complement and C3 deposition for opsonophagocytic killing [23]. Conversely, shorter pneumococcal chains provide a competitive advantage to support evasion of host immune barriers [24]. For *S. mutans*, longer chains appear to interfere with phagocytosis by host immune cells [23,25]. In terms of conditions that promote long-chaining in a host, increased chain length in streptococci has been linked to exposure to antisera for group A streptococcus [26]. Streptococcal chain length can also vary due to gene mutations that result in incomplete cleavage of peptidoglycan between daughter cells, altered expression of proteins that mediate cell separation, or mutations in TCSs [22,23, 33,36]. CdhA, for example, is essential for formation of long chains in group A streptococcus [34] and PcsB is important for cell division in *Streptococcus* spp.; an absence of PcsB in GBS leads to defective cell growth and division, although a role in cell separation is unclear [35]. Mutations in TCSs in *S. sanguinis* result in longer chains [22]. The observation that multiple TCSs are associated with chaining suggests that these systems might offer a degree of redundancy to ensure correct cell separation at appropriate times and in conditions that can be sensed and responded to in a controlled way. The activity of genes that govern cell separation (some of which are part of TCS regulons) during infection would be of interest to understand the conditions that promote long-chaining in a host, and how such phenotypes influence pathogenesis.

In summary, this study shows that altered chain length in GBS can affect population size estimates based on c.f.u. measures and can affect GBS virulence in experimental models. For strains that form longer chains, disruption of GBS chains increases c.f.u. recovery from *in vitro* assays. The genes involved in chain formation in GBS and the impact of longer chains on other aspects of virulence such as adhesion, phagocytosis, and intracellular survival remain areas for future investigation.

## supplementary material

10.1099/mic.0.001453 Uncited Fig. S1.

## Abbreviations

c.f.u.: colony forming units
Cm^R^: chloramphenicol-resistant
GAPDH: glyceraldehyde 3-phosphate dehydrogenase
GBS: group B streptococcus
p.i.: post-infection
Sp^R^: spectinomycin-resistant
TCS: two-component system
Ts: temperature-sensitive
