# Supplementary material for: Alterations in cell arrangements of group B streptococcus due to virulence factor expression can bias estimates of bacterial populations based on colony count measures
Source: Microbiology (Reading). 2024 Apr 24;170(4):001453. doi: 10.1099/mic.0.001453 (PMC11084685; doi:10.1099/mic.0.001453)

Alterations in cell arrangements of group B streptococcus due to virulence factor expression can bias estimates of bacterial populations based on colony count measures.

Ruby Thapa[1*], Kelvin G. K. Goh[1*], Devika Desai[1], Ellen Copeman[1], Dhruba Acharya[1], Matthew J. Sullivan[1,2], and Glen C. Ulett[1†]

[1]School of Pharmacy and Medical Sciences, and Menzies Health Institute Queensland, Griffith University, Gold Coast Campus, QLD, Australia 4222

[2]School of Biological Sciences, University of East Anglia, Norwich, NR4 7TJ, United Kingdom

*Equal contributions.

SUPPLEMENTAL MATERIAL

List of items:

Supplemental Fig. 1. Culture densities of 874391, 874931p*gapC*, and 874391pDL278. The OD600 absorbance readings of overnight cultures from three strains measured pre-disruption were similar (n = 10).

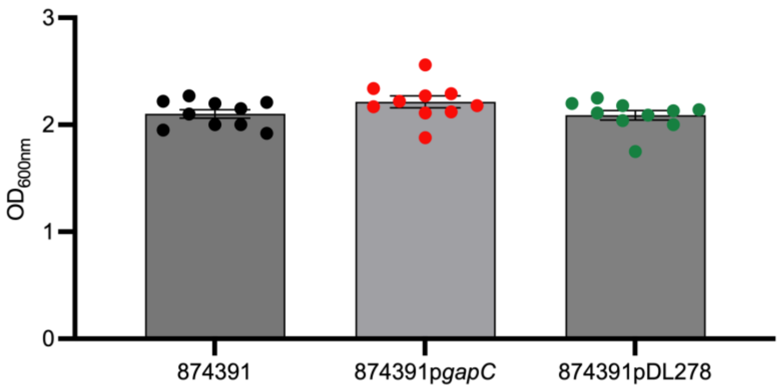

Supplement: Uncited Fig. S1. [file mic-170-01453-s001.pdf]
